# Identification of a Chlorophyll-Deficient Mutant in Maize Associated with Exogenous Vector Insertion

**DOI:** 10.3390/plants15020266

**Published:** 2026-01-15

**Authors:** Wenqi Zhou, Haoyue Wang, Chunxia Liang, Haijun He, Yongsheng Li, Xiaorong Lian, Xiaojuan Wang, Xiaoyun Dong, Zengke Ma, Zhongxiang Liu, Yuqian Zhou

**Affiliations:** Maize Research Center of Gansu Province, Crop Research Institute, Gansu Academy of Agricultural Sciences, Lanzhou 730070, China; 13008772771@163.com (H.W.); 18776966303@163.com (C.L.); hhj007@sina.com (H.H.); lys087@163.com (Y.L.); lianxr@126.com (X.L.); wangxj839@sina.com (X.W.); 13919361778@163.com (X.D.); zengkema@sina.com (Z.M.); lzhxiang@sina.com (Z.L.)

**Keywords:** maize (*Zea mays*), leaf color, chlorophyll-deficient, foreign vector, co-segregation

## Abstract

Leaf color mutants are commonly characterized by altered chlorophyll content and aberrant chloroplast development, making them valuable models for investigating photosynthetic mechanisms and chloroplast biogenesis. In this study, an albino mutant was isolated from a population of transgenic maize breeding lines. Genetic analysis indicated that the mutant phenotype is inherited in a Mendelian manner and is controlled by a single nuclear locus. This was supported by a *χ*^2^ test performed on the T_2_ generation, which confirmed a segregation ratio consistent with 3:1 (176:68, *χ*^2^ = 1.07 < *χ*^2^_0.05_ = 3.84, *p* > 0.05). Microscopic examination revealed the absence of normally developed chloroplasts in mutant cells. Further expression analysis of chloroplast genes via Northern blotting and quantitative real-time PCR (qRT-PCR) suggested that the mutation impairs the regulation of plastid-encoded polymerase (PEP)-dependent chloroplast gene expression. Notably, PCR-based co-segregation analysis indicated that the mutant phenotype is associated with the entire inserted vector sequence, rather than a point mutation or a small genomic deletion. In conclusion, this paper reports the isolation and phenotypic characterization of an etiolated mutant from a transgenic maize breeding population, including comparative ultrastructural analysis of chloroplasts, co-segregation validation, and chloroplast gene expression profiling. These results enhance our understanding of the physiological and molecular mechanisms underlying chlorophyll-deficient mutations in plants.

## 1. Introduction

The leaf is the principal organ of photosynthesis and is critical for determining photosynthetic efficiency, thereby influencing plant growth, development, and yield formation [1]. Chlorophyll-deficient mutants are valuable genetic resources for identifying genes responsible for mutant traits and elucidating gene functions and regulatory mechanisms, and have consequently become a major research focus in plant biology [2]. The models for photosynthesis serve as well-established systems for investigating photosynthetic mechanisms and chloroplast development [3,4,5], enabling investigations into chloroplast ultrastructure, the biosynthesis and degradation of photosynthetic pigments, and the expression and regulation of associated genes [1]. Additionally, they serve as powerful systems for analyzing chlorophyll metabolism and related pathways in plants [6].

Leaf color mutations can be induced by various factors that directly or indirectly affect pigment accumulation. The primary mechanisms frequently involve mutations in genes associated with chlorophyll biosynthesis or degradation [7,8,9,10], chloroplast development and function, or photomorphogenesis [11,12]. The most conspicuous phenotype of these mutants is an alteration in leaf pigmentation, which is typically most pronounced in the seedling stage [13]. Early studies classified chlorophyll-deficient mutants into five categories based on phenotypic observations regarding appearance, namely an albino appearance, yellowing, a pale green appearance, a variegated appearance, and a striped [14]. Subsequently, this classification was expanded to eight types: yellowing, green-yellow, yellow-green, pale green, albino, white-green, green-white, and striped [15].

To date, more than 200 genes or quantitative trait loci (QTLs) associated with leaf color phenotypes have been identified or cloned in maize, with most mutations localized to specific chromosomes. Among these, 23 well-characterized maize leaf color mutation genes have been precisely mapped, distributed across all chromosomes except chromosome 4 [2]. Notable examples of maize chlorophyll-deficient mutants include the light-insensitive *elongated mesocotyl 1* (*elm1*) mutant, which exhibits a pale green leaf phenotype and encodes a 34 kD protein consisting of 297 amino acids that shares 50% sequence identity with AtHY2. *elm2* displays an abnormal leaf color caused by a 21 bp deletion, while other related mutants such as *ygl1* (*yellow-green leaf 1*), *vyl* (*virescent yellow leaf*), and *nec-t* (*yellow leaf*) also exhibit altered chlorophyll accumulation [16]. *elm1*: A nuclear gene-controlled pale green leaf mutant characterized by pale green leaves, reduced photosynthetic pigment content, and decreased photosynthetic efficiency. *elm2*: A nuclear gene-controlled yellow-green leaf mutant exhibiting abnormal chloroplast structure and impaired photosynthetic pigment synthesis. *ygl1*: A nuclear gene-controlled yellow-green leaf mutant caused by a mutation in the chlorophyll synthase gene, leading to reduced chlorophyll synthesis. *vyl*: A nuclear gene-controlled yellow leaf mutant with abnormal chloroplast development and significantly reduced photosynthetic pigment content. These mutants provide valuable insights into the genetic and physiological mechanisms underlying leaf color variation and chloroplast function.

In green plants, the plastid originating from cyanobacterial endosymbiosis is under dual genomic control by both nuclear and plastid genomes [17]. Chloroplast gene transcription is mediated by two distinct RNA polymerases (RNAPs): a nuclear-encoded phage-type RNA polymerase (NEP) and a plastid-encoded RNA polymerase (PEP) derived from cyanobacteria [14,18,19,20]. While NEP predominantly transcribes housekeeping genes (e.g., *RPO genes*, *RRN16*, *CLPP1*) during early plastid development, PEP becomes dominant in later stages, driving the expression of photosynthesis-related genes essential for thylakoid membrane biogenesis and photosynthetic machinery assembly [15,21]. The transcriptional activities of NEP and PEP are coordinately regulated by endogenous and environmental signals, including light, temperature, circadian rhythms, and redox status [22]. Notably, PEP—a prokaryotic-type multisubunit enzyme—requires nuclear-encoded auxiliary subunits (PAPs/pTACs) for its assembly and functionality. These auxiliary proteins, such as PAP2/pTAC2 and PAP3/pTAC10, interact with PALECRESS (PAC) to modulate PEP complex stability and activity [23]. PEP activity is further fine-tuned by sigma factors (SIGs), which are nuclear-encoded transcription initiation factors whose expression and activity are tightly controlled at transcriptional and post-translational levels [24].

In this study, we identified a novel maize chlorophyll-deficient mutant exhibiting disrupted chloroplast development and seedling lethality in the three-leaf stage. PCR-based co-segregation analysis linked the mutant phenotype to the insertion of a foreign vector sequence, suggesting that PEP dysfunction—potentially through interference with PAP/pTAC-mediated complex assembly—underlies the observed developmental defects. This mutant provides a unique model for investigating PEP’s role in chloroplast biogenesis and plant survival.

## 2. Results

### 2.1. Phenotypic Characterization of the Chlorophyll-Deficient Mutant (MT)

**Figure 1:**
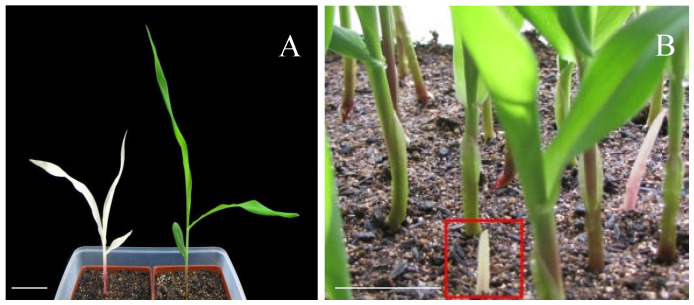
Phenotypes of the mutant (MT) and wild-type (WT) plant. (**A**) The albino mutant (left) and the control (right); (**B**) the non-leaf phenotype of mutant seedlings. Heterozygous plants isolated homozygous phenotypes of albino seedlings. Bars = 5 cm.

A maize *chlorophyll-deficient mutant* (MT) was isolated from a transgenic population. The mutant exhibited a distinct phenotype compared to wild-type (WT) plants, characterized by albinism in seedling leaves and significantly reduced plant height (Figure 1A). This phenotypic variation is likely attributable to defects in chlorophyll biosynthesis and deficiencies in other essential photosynthetic pigments, resulting in compromised photosynthetic capacity. In contrast to WT plants, which retain normal green pigmentation and autotrophic growth through photosynthesis, the mutant seedlings displayed lethal albinism under autotrophic conditions. The albino seedlings (highlighted by red boxes) showed a striking phenotypic contrast against the surrounding green WT plants (Figure 1B).

The chlorophyll a and b contents in the third leaves of both mutant and WT plants were quantitatively determined. Compared with the WT, both pigments were significantly reduced in the chlorophyll-deficient mutant. Specifically, chlorophyll a content was 2.32 mg/g in the WT and only 0.13 mg/g in the MT, representing merely 5.6% of the WT level (*p* < 0.01). Similarly, chlorophyll b content decreased from 0.61 mg/g in the WT to 0.02 mg/g in the MT, amounting to just 3.3% of the control value (*p* < 0.01). Albino seedlings, which exhibit a near-complete absence of chlorophyll, are incapable of performing effective photosynthesis and thus cannot sustain autonomous growth. These seedlings eventually die once the seed-reserved nutrients are depleted. The observed albinism is attributed to genetic mutations that disrupt key pathways of pigment biosynthesis, thereby severely impairing the plant’s ecological fitness under natural conditions. Notably, mutant seedlings exhibited developmental defects during germination, including delayed leaf emergence and transient etiolation, implying that the mutation disrupts early germination processes.

### 2.2. The Albino Mutant Exhibits Arrested Chloroplast Development in an Early Biogenesis Stage

**Figure 2:**
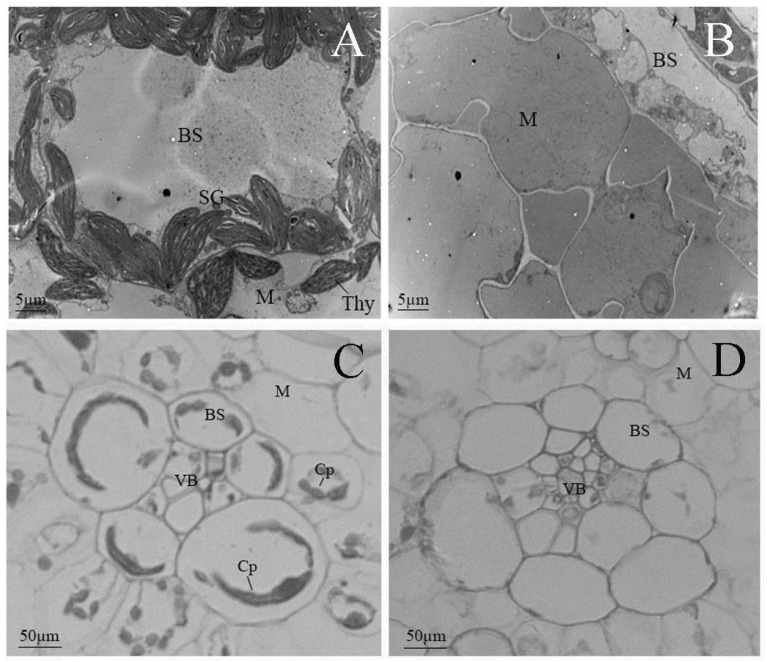
Microscope observation of chloroplast structure in mutant and wild-type plants in an early biogenesis stage. (**A**,**B**) Transmission electron microscopic images; bars: 5 µm. (**C**,**D**) Light microscopic images; bars: 50 µm. (**A**,**C**) Wild-type; (**B**,**D**) mutant-type. Abbreviations: Cp, chloroplast; SG, starch grain; Thy, thylakoid lamellar; BS, bundle-sheath cells; M, mesophyll cells; VB, vascular bundle.

To investigate the developmental variation of chloroplasts, we compared the chloroplast ultrastructure of the mutant with that of the wild-type using transmission electron microscopy (TEM). In wild-type plants, mature chloroplasts within Kranz anatomy cells exhibited well-defined structures, including a distinct matrix (M) and an organized system of thylakoid membranes (Thy). These thylakoids were stacked into grana (G), which are critical for photosynthetic function. Additionally, starch granules (SGs), which serve as transient storage for photosynthetic products, were readily observed (Figure 2A). For electron microscopy observation, the tissue sample was taken from the apical sections of the second leaf from the top down.

By contrast, the albino mutant displayed severe defects in chloroplast development in the same development stage. No typical chloroplast structures were discernible; instead, the organelles exhibited aberrant morphology characterized by a relatively homogeneous matrix and a markedly underdeveloped internal membrane system. Electron-lucent stroma with a complete absence of organized thylakoid membranes and grana and mitochondrial structures, typically characterized by compact cristae, were also poorly defined. These observations indicate a failure in the formation of functional photosynthetic architectures (Figure 2B).

Light microscopy further corroborated these findings. Wild-type mesophyll cells contained regularly shaped, distinct round or oval chloroplasts, clearly visible within both vascular bundle sheath cells (BSCs) and mesophyll cells (MCs). In the mutant, however, chloroplasts were irregular in size and shape, lacking defined structural features, which can be attributed to the severe deficiency in chlorophyll and other essential photosynthetic pigments (Figure 2C,D).

Collectively, these findings indicate that chloroplast development in the mutant is disrupted and halted in an early stage of biogenesis, which leads to non-functional plastids that are incapable of supporting photosynthesis.

### 2.3. Expression of Chloroplast Genes in the Mutant

To assess the effect of the mutation on the expression of photosynthetic genes encoded in the chloroplast, we analyzed the transcript levels of selected genes in mature leaves of wild-type and mutant seedlings using Northern blotting and quantitative RT-PCR (qRT-PCR). Based on prior research findings, the genes *psbA*, *psbH*, *psaJ*, *trnV*, and *rbcL* are recognized as key components of the plastid-encoded chloroplast genome (PEP; Class I genes) and are primarily involved in photosynthesis-related processes, as well as *clpP* and *rpoB*, which depend on the nucleus-encoded polymerase (NEP; Class III genes) [25,26]. Consequently, these genes are frequently selected as molecular markers for evolutionary relationship analyses and functional genomics studies.

**Figure 3:**
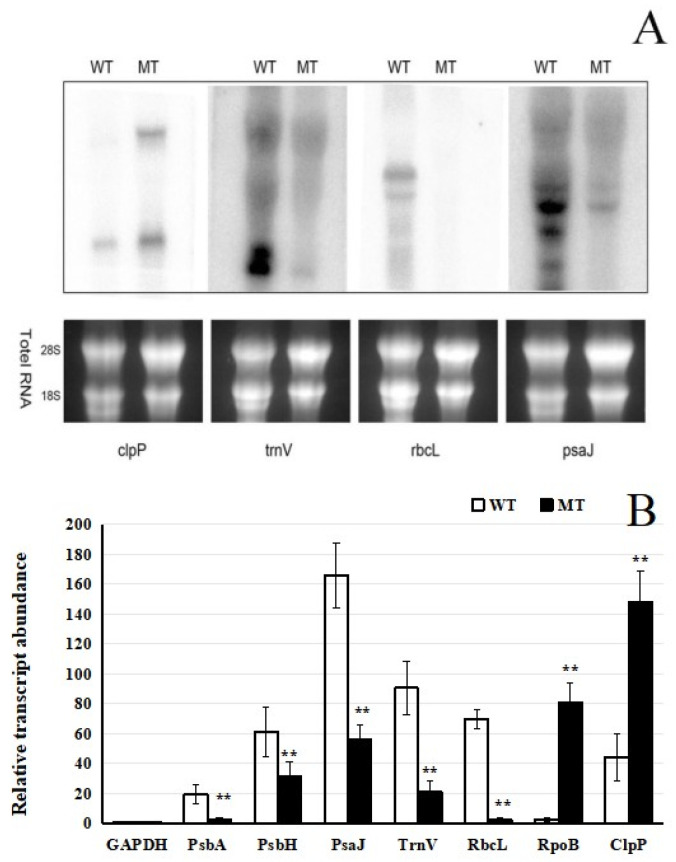
Expression analysis of chloroplast genes. (**A**) RNA gel-blot hybridization assays of selected chloroplast genes in mutant and wild-type plants. An amount of 10 µg of total RNA was loaded per lane. (**B**) qRT-PCR expression analysis of selected chloroplast genes in mutant and wild-type plants. Total RNA was extracted from mature leaves. The average of three technical replicates is plotted, and error bars represent the standard deviations of triplicates. ** indicates significant differences, indicated by *p* < 0.01.

Northern blot analysis revealed substantial differences in transcript accumulation between wild-type (WT) and mutant (MT) plants for several genes, including *trnV*, *rbcL*, *psaJ*, *psbA*, and *psbH*. In WT samples, these genes showed strong hybridization signals, whereas in MT samples, the signals were markedly reduced or absent, indicating severe transcriptional suppression or complete loss of expression. Notably, the expression of *clpP* and *rpoB* was barely detectable in the WT, whereas the relative transcript levels were significantly higher in the MT (Figure 3A,B). These findings were further validated by qRT-PCR, which confirmed the significant downregulation or absence of transcript accumulation for the affected genes in the mutant background. The relative expression levels of several genes (*psbH*, *psbA*, *clpP*, *rpoB*, *trnV*, *rbcL*, and *psaJ*) were compared between WT and MT samples. Consistent with the Northern blot results, the relative expression levels of *trnV*, *rbcL*, *psaJ*, *psbA*, and *psbH* in the mutant were significantly lower than those in the control (*p* < 0.05). However, the expression levels of *clpP* and *rpoB* genes were significantly upregulated in the MT (Figure 3A,B). The selected internal reference genes remain unchanged.

Collectively, both analytical methods demonstrated a substantial decrease in the expression of specific genes in the mutant, indicating that the mutation may disrupt transcriptional regulation within plastids. In particular, expression of PEP-dependent genes was notably reduced, while that of NEP-dependent genes was elevated, as supported by both Northern blot and qRT-PCR analyses (Figure 3A,B). This reciprocal regulation of PEP- and NEP-dependent genes suggests a loss of coordinated expression between the plastid- and nuclear-encoded transcription systems, which likely contributes to the observed defects in chloroplast development and function.

### 2.4. Association Between Mutant Phenotype and Transgenic Vector

**Figure 4:**
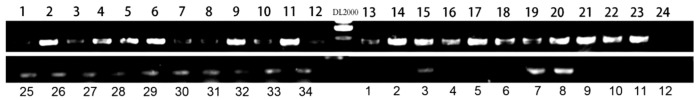
PCR-based co-segregation analysis. Result for >1678F/2090R.

**Figure 5:**
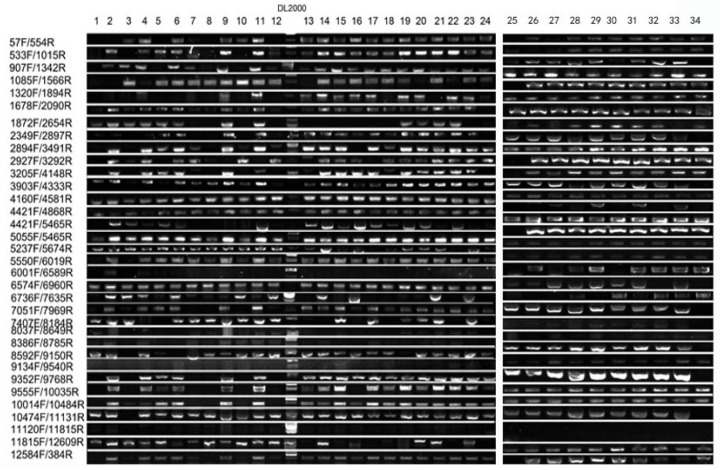
Co-segregation analysis for mutant type.

**Figure 6:**
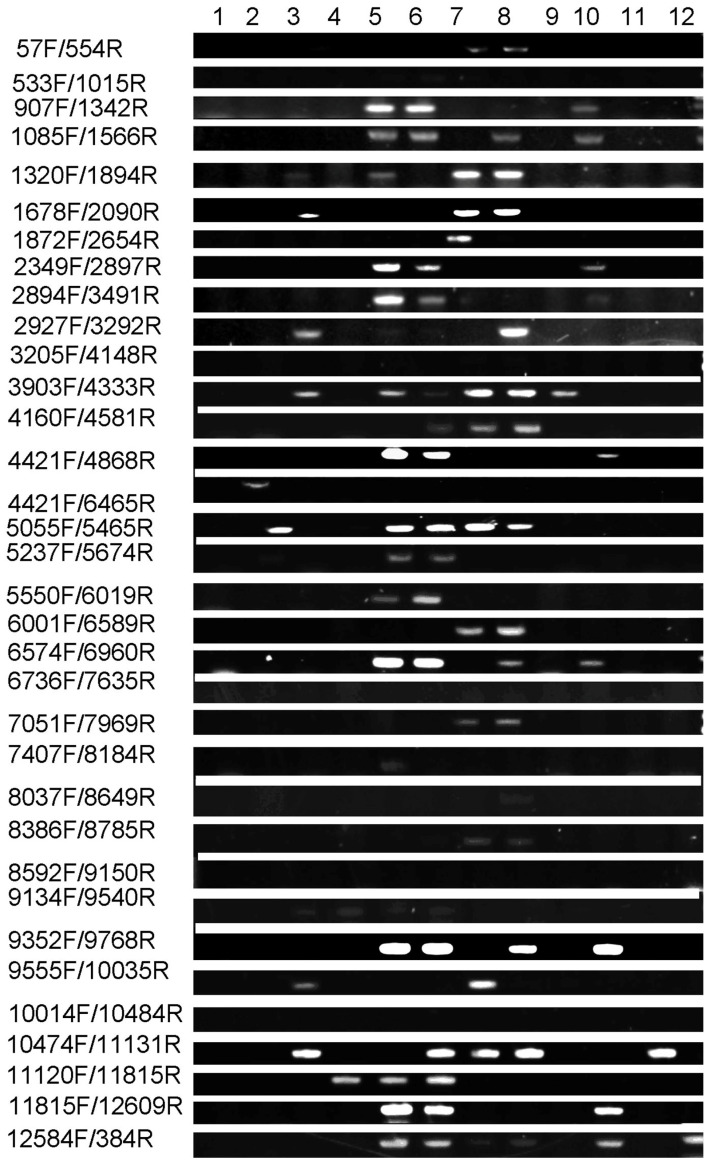
Co-segregation analysis for wild-type.

Given the transgenic origin of the mutant, we aimed to determine whether the observed albino phenotype was directly caused by the integration of T-DNA from the transgenic vector. To address this, we performed a comprehensive PCR-based co-segregation analysis targeting the full 12 kb transgenic vector. The construct was systematically divided into 34 overlapping DNA fragments, and specific primer pairs were designed to amplify each region across the entire sequence (Table 1). Genomic DNA from 34 albino individuals in the T_3_ generation and 12 phenotypically wild-type siblings was screened for the presence or absence of each fragment. The results demonstrated complete genetic linkage: all 34 albino plants retained every segment of the intact transgene, whereas no T-DNA signal was detected in any of the wild-type individuals (Figure 4 and Figure 5). This absolute co-segregation of the transgenic insert with the albino phenotype—without any recombination events observed in the progeny—strongly supports a causal relationship (Figure 5 and Figure 6). Moreover, the consistent absence of the transgene in all normally pigmented plants indicates that the T-DNA insertion is not merely genetically linked but likely disrupts a gene essential for chloroplast development or chlorophyll biosynthesis. These findings provide robust molecular evidence that the integration of the 12 kb transgenic vector constitutes the causative genetic lesion responsible for the albino phenotype.

### 2.5. Chi-Square Analysis Showed That the Chloroplast-Deficient Phenotype Is Caused by a Recessive Nuclear Mutation in a Single Gene

Genetic analysis indicated that the mutant phenotype is inherited in a Mendelian manner and is controlled by a single nuclear locus. This was supported by a *χ*^2^ test conducted on the T_2_ generation, which confirmed a segregation ratio consistent with a 3:1 ratio of WT/MT (176:68, *χ*^2^ = 1.07 < *χ*^2^_0.05_ = 3.84, *p* > 0.05). Genetic segregation analysis indicated that the segregation ratio between the wild-type and mutant was 3:1, consistent with Mendel’s law of monogenic recessive inheritance. To characterize the genetic basis of the mutant, a T_2_ population was derived from a cross involving A188 × B73 and Zheng58. All T_1_ plants exhibited the WT phenotype. In the T_2_ generation, phenotypic segregation ratios (176 wild-type: 68 mutant; *χ*^2^ = 1.07 < *χ*^2^_0.05_ = 3.84, *p* > 0.05) were consistent with a 3:1 ratio, suggesting control by a single recessive locus. This segregation pattern was maintained in the T_3_ generation (226 wild-type: 57 mutant; *χ*^2^ = 3.56, *p* > 0.05). Statistical analysis of segregation patterns in T_2_ and T_3_ hybrid generations, coupled with chi-square testing, revealed that the chlorophyll-deficient mutant is governed by nuclear single-gene recessive inheritance. Consequently, the candidate gene was preliminarily mapped through an integrated approach that combines bulked segregant analysis sequencing (BSA-seq) with bulked segregant RNA sequencing (BSR-seq).

### 2.6. The BSR-Seq Was Used to Identify Candidate Genes

The BSR-Seq revealed that a total of 20,345 genes were expressed in WT plants and 22,377 genes in the MT, with an expression threshold of RPKM ≥ 1. By applying stringent criteria of fold change > 4 and FDR < 0.001, we identified 944 differentially expressed genes, including 780 up- and 164 downregulated genes in the mutant compared to the wild-type.

**Figure 7:**
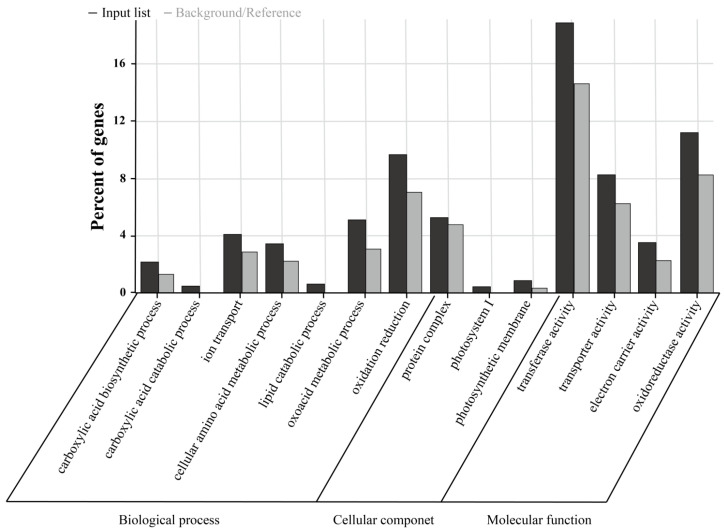
GO enrichment analysis.

GO enrichment analysis was conducted on the 2-fold differentially expressed genes in the samples (Figure 7). In the biological process category, the differentially expressed genes were mainly enriched in functional items such as oxidation–reduction, ion transport, and the metabolism of some small-molecule compounds, including amino acids, lipids, oxoacids, and carboxylic acids. In the cellular component category, the differentially expressed genes were significantly enriched in functional items such as photosynthetic membranes, photosystem I, and protein complexes. At the molecular function level, the differentially expressed genes were significantly enriched in functional items such as transferase activity, oxidoreductase activity, transporter activity, and electron carrier activity.

The BSR-Seq approach was employed to map the mutant gene. During this process, more than 340,000 SNP sites/InDel were identified by comparing the MT and WT sample pools. Further, using a classic Bayesian algorithm, the candidate gene was localized to a genomic interval of approximately 900 kb (13.0–13.9) on chromosome 5 of maize. Combining fine mapping and transcriptome sequencing, GO analysis and functional validation were used to screen and verify the candidate genes.

## 3. Discussion

### 3.1. Effects of Chlorophyll-Deficient Mutations on Plastid Transcription

Chloroplast biogenesis requires coordinated gene expression mediated by two RNA polymerases: the nuclear-encoded polymerase (NEP) and the plastid-encoded polymerase (PEP). NEP predominantly transcribes housekeeping genes during early chloroplast development, whereas PEP serves as the major RNA polymerase in mature chloroplasts [27,28]. Both enzymes remain active in non-green plastids and chloroplasts throughout development, with their transcriptional activities modulated by diverse endogenous and exogenous factors. This regulation necessitates precise coordination with both plastid and nuclear gene expression programs.

High-resolution cryo-electron microscopy structures of the chloroplast PEP complex and its transcription elongation complex have been determined. This study elucidated the subunit composition and assembly mechanisms underlying the chloroplast transcriptional machinery [24]. The study demonstrated that chloroplast PEP contains a unique functional module encoded by nuclear genes, translated in the cytoplasm, and imported into the chloroplast. This nuclear-encoded module is essential for PEP catalytic activity, underscoring the role of nuclear control in plastid gene expression. These structural insights advance our understanding of chloroplast transcription and the regulatory crosstalk between NEP and PEP, particularly under genetic perturbation. Further investigations using the *var6-1* mutant, which exhibits a “flower spot” leaf phenotype, demonstrated that the reduced accumulation of the PEP complex in *var6-1* directly results from misregulation of PALICRESS expression. This finding reveals a previously unknown role for PALICRESS in regulating PEP complex stability [23].

In the Paseno Project [29], a *chrysanthemum yellow-green striped* mutant (*cmvv*) was utilized to examine the function of the *RPOC2* gene. Loss of *RPOC2* activity disrupted chloroplast biogenesis and thylakoid membrane formation by downregulating the transcription of 28 chloroplast genes. Among these, 9 genes were under exclusive PEP promoter control, 1 contained both PEP and NEP promoters, and 17 were regulated solely by NEP promoters. These results demonstrate that the *rpoc2* mutation specifically compromises PEP functionality, resulting in the downregulation of PEP-dependent genes while exerting minimal impact on NEP-dependent transcription. This underscores the distinct but coordinated contributions of NEP and PEP in chloroplast gene expression.

Studies on the rice chloroplast-deficient mutant *wsl887* indicate that the mutation severely impairs PEP transcriptional activity, thereby disrupting normal chloroplast development [30]. Similarly, the pdml mutant exhibited a sharp decline in transcripts from PEP-dependent chloroplast genes, accompanied by increased accumulation of NEP-dependent transcripts, further highlighting the essential role of PEP in maintaining plastid function [31]. Alterations in chloroplast gene expression are shown in Figure 3. Northern blot and qRT-PCR analyses were conducted to compare the expression of photosynthesis-related genes in mature leaves of WT and MT seedlings. The results indicated a substantial reduction in transcript levels of specific genes in the mutant relative to the wild-type. Numerous studies have documented that mutations impairing chloroplast function often lead to downregulation of class I genes (primarily transcribed by PEP), while class III genes (exclusively transcribed by NEP) are upregulated [32,33,34]. For example, in the *NIL-PDDOL* mutant, chloroplast gene expression profiling revealed decreased mRNA abundance of class I genes and a marked increase in class III gene expression. Functional characterization of the PDM1 protein in *Arabidopsis* further demonstrated that PEP-dependent chloroplast transcripts were severely reduced in *pdm1* mutants, whereas NEP-dependent transcripts accumulated to higher levels [29,35].

In summary, mutations affecting chloroplast transcription involve disrupted coordination between NEP and PEP activities, as well as alterations in specific gene expression patterns. These transcriptional defects are frequently associated with loss of PEP function, which impairs chloroplast development and overall plastid functionality.

### 3.2. Mutant Phenotype Linked to the Entire Foreign Vector Sequence

Interestingly, the mutant phenotype is associated with the insertion of an intact exogenous vector sequence, rather than resulting from point mutations or small-scale insertions or deletions. This conclusion was corroborated by a blue-white screening assay [36,37], which confirmed that the phenotype is linked to the presence of the full-length foreign DNA sequence. This finding is consistent with previous reports, such as the identification of the *CSP41b* gene in chloroplasts, which influences leaf coloration in the rice vein yellowing mutant *yml*, as well as the cloning of the maize yellow leaf gene *ZmNPPR5* [36,38]. While the blue-white screening assay established the correlation between vector insertion and mutant phenotype, studies in rice have emphasized genetic mapping and the role of specific genes in leaf color determination, and research in maize has focused on the molecular cloning of yellow leaf genes. By contrast, the present study investigates luteinization-related mutations in maize, providing further insight into the relationship between exogenous DNA integration and chloroplast development.

The mutant was identified from a transgenic population. To investigate whether the mutant phenotype was associated with the insertion of the foreign vector, the transgenic vector was introduced via particle bombardment, and the T_3_ generation was subjected to molecular analysis. This population consisted of 12 wild-type and 34 mutant seedlings. The 12 kb exogenous vector was isolated from both mutant and wild-type plants for comparative examination. The full-length vector was detected in all 34 mutant seedlings but was absent in all 12 wild-type individuals, indicating that the chlorophyll-deficient phenotype is tightly linked to the presence of the intact transgenic vector. It is plausible that the insertion of the vector leads to reduced expression or functional disruption of key genes involved in chlorophyll biosynthesis or chloroplast development. These results strongly suggest that the mutant phenotype is directly caused by the integration of the complete exogenous vector sequence.

These findings underscore the complexity of interactions between exogenous DNA insertion and phenotypic consequences, emphasizing the importance of analyzing intact vector sequences rather than concentrating exclusively on single-nucleotide polymorphisms or small DNA fragments. This study establishes a critical foundation for understanding how large DNA constructs influence agronomically relevant traits and provides a framework for further investigating the molecular mechanisms driving such phenotypic alterations.

### 3.3. The BSA (Bulked Segregate Analysis-Sequencing) and BSR Methods Were Used to Identify Candidate Genes

The four primary approaches for applying BSA and BSR technologies are MutMap, MutMap+, MutMap-Gap, and QTL-seq [39,40,41]. These methods share a common underlying principle: for a given target trait, parental lines exhibiting pronounced phenotypic divergence are selected to generate a segregating population. From this population, a subset of individuals displaying extreme phenotypes for the trait of interest is chosen and pooled to construct two DNA bulks. Whole-genome sequencing is subsequently performed on these bulks. Typically, genomic DNA from both parental lines is also sequenced and used as a reference to ensure accurate interpretation of the results. By comparing sequence variations between the two DNA pools, genomic regions exhibiting significant allele frequency differences are identified as candidate intervals, which likely harbor the causal gene or quantitative trait loci associated with the target trait.

## 4. Materials and Methods

### 4.1. Plant Materials and Growth Conditions

The maize mutant was initially isolated from a transgenic breeding line. The foreign vector PCAMBIA3300-cry1C* was introduced via particle bombardment into the embryogenic callus of the A188 × B73. Pollen from the T_0_-positive individual was subsequently crossed with Zheng58 to generate the T_2_ population, where albino phenotypes were observed in the T_2_ generation. Plants were cultivated at the ZhangYe experimental station of the Gansu Academy of Agricultural Sciences, whereas plants used for DNA, RNA, and microscopy analyses were grown in a growth chamber under a 16 h light (28 °C)/8 h dark (23 °C) cycle. The seedlings were grown in a substrate consisting of nutrient soil and vermiculite mixed at a volume ratio of approximately 1:1, and the containers were covered with plastic film to minimize water evaporation. Upon reaching the second-leaf stage, wild-type and mutant individuals were phenotyped and subjected to genetic analysis. Tissue samples were collected when the third leaf began to emerge from the whorl (approximately 12 days after planting).

### 4.2. Microscopy Analysis

Leaf samples from wild-type and mutant plants were prepared for transmission electron microscopy (TEM). All leaf tissues were dissected into approximately 1 mm^2^ segments and fixed in 2.5% glutaraldehyde in 0.1 mol/L phosphate buffer (PBS, pH 7.2). To preserve ultrastructural integrity, samples were handled under conditions ensuring freshness and representativeness. Initial screening at low magnification was performed to evaluate overall cellular architecture, followed by detailed examination at higher magnifications to resolve subcellular features. Imaging parameters, including magnification, contrast, and brightness, were optimized to enhance clarity. Acquired images were recorded and subsequently analyzed through qualitative and quantitative measures in accordance with established morphological criteria.

For light microscopy, tissue processing involved sectioning and staining. An initial low-magnification assessment was conducted to identify regions of interest, which were then centered and examined under higher magnification. Fine focusing was applied to achieve optimal resolution of cellular morphology and tissue organization [5,42]. Apical sections of the second leaf were used as electron microscope observation materials.

### 4.3. DNA Extraction

1. Preparation of plant materials: Fresh leaves were selected, rinsed with distilled water to remove surface contaminants, and air-dried to remove excess moisture. The tissue was placed in a pre-cooled mortar, frozen with liquid nitrogen, and ground into a fine powder, which was transferred to a centrifuge tube for further processing. 2. CTAB extraction: Preheated CTAB buffer (2%, 65 °C) was added to powdered tissue and gently inverted. The mixture was gently inverted and incubated at 65 °C for efficient cell lysis, with periodic inversion to enhance extraction. An equal volume of chloroform-isopropanol (24:1) was added to denature proteins. After mixing by gentle inversion and centrifugation, phases separation took place (DNA in the upper aqueous layer; denatured proteins in the middle and organic phase). The aqueous layer was carefully transferred to a new tube. 3. DNA precipitation and washing: Either 2/3 or an equivalent volume of pre-cooled isopropanol or anhydrous ethanol was added to the aqueous solution to precipitate DNA under high-salt conditions. The tube was gently inverted, forming a foamy DNA precipitate. After centrifugation, the supernatant was discarded. The pellet was washed with 70% ethanol to remove salts and impurities and centrifuged again, before residual ethanol was removed by aspiration. The DNA pellet was dried at room temperature or under vacuum. 4. DNA dissolution and detection: An appropriate volume of TE buffer or nuclease-free water was added to dissolve the DNA. The purity and concentration were assessed by spectrophotometry and agarose gel electrophoresis.

### 4.4. RNA Extraction

1. Total RNA was isolated from leaves using the TRIzol reagent (Takara) in steps that include cell or tissue fragmentation. For tissue samples, an appropriate amount of tissue was taken into a mortar, liquid nitrogen was added, and the tissue was quickly ground into powder. The powder was then transferred to a centrifuge tube containing TRIzol reagent and mixed thoroughly. After TRIzol addition and sufficient lysis, the nucleoprotein complex was completely dissociated by allowing the mixture to stand at room temperature for 5 min. Chloroform was then added, and the mixture was vigorously oscillated for 15 s, followed by standing at room temperature for 2–3 min. The mixture was then centrifuged at 4 °C and 12,000× *g* for 15 min, at which point the solution separated into three layers: the upper, colorless water phase containing RNA; the middle, white protein layer; and the lower, red organic phase containing DNA and lipids. 2. RNA precipitation was performed. The upper water phase was carefully absorbed and transferred to a new centrifuge tube, and an equal volume of isopropanol was added. The mixture was mixed well and left at room temperature for 10 min to precipitate the RNA. After centrifugation at 4 °C and 12,000× *g* for 10 min, white or transparent RNA pellets appeared at the bottom of the tube. For RNA washing, the supernatant was discarded, and 75% ethanol (prepared with DEPC-treated water) was added to wash the RNA pellet. The tube was then vortexed or gently inverted, and centrifuged at 4 °C, 7500× *g* for 5 min, and the supernatant discarded. Any residual ethanol was sucked out, and the RNA pellet was dried at room temperature or under vacuum. 3. RNA was dissolved. An appropriate amount of water or RNAase-free slow infusion was added to the dried RNA pellet, gently blown, or swirled to dissolve the RNA completely. The RNA solution could then be stored at −80 °C.

### 4.5. Quantitative Real-Time PCR Analysis (qRT-PCR)

Real-time PCR amplification was performed on a Bio-Rad CFX96 Real-Time System in accordance with the manufacturer’s instructions. Actin was employed as the reference gene for normalization, using the following primers: forward primer (TACGAGATGCCTGATGGTCAGGTCA) and reverse primer (TGGAGTTGTACGTGGCCTCATGGAC). All qRT-PCR experiments included three technical replicates. Gene expression analysis was performed using the 2^−^^△△^*^C^*^T^ method according to Livak and Schmittgen [43]. Data collection: *C*T values for target and reference genes were obtained; Δ*C*T calculation: Δ*C*T = *C*T (target) − *C*T (reference); ΔΔ*C*T determination: ΔΔ*C*T = Δ*C*T (sample) − Δ*C*T (control); relative quantification: expression levels were calculated as 2^−ΔΔ^*^C^*^T^.

### 4.6. Statistical Analysis

Data were analyzed using *t*-tests or ANOVA, with results visualized through bar graphs showing relative expression levels and standard errors.

### 4.7. RNA Gel-Blot Hybridization Assays

Total RNA (10 μg) was transferred to nylon membranes and hybridized with ^32^P-labeled cDNA probes. Following stringent washing, blots were exposed to *X*-ray film. Probe details were as described by Udy et al. [44].

### 4.8. Co-Segregation Analysis

The PCAMBIA3300-cry1C* vector sequence (Supplementary Table S1) was divided into segments for PCR amplification to assess its distribution in the T_3_ generation. Marker sequence information is provided in Table 1.

### 4.9. BSR Sequencing Data Statistics

Thirty wild-type plants and thirty mutant plants were selected, and RNA was extracted from each individual plant. Two mixed sample pools—one for the wild-type and one for the mutant—were constructed either by pooling RNA after individual extraction or by mixing based on quantitative concentration for RNA-Seq analysis [45]. The resulting clean reads numbered 32,638,200 and 34,401,500 for the wild-type and mutant samples, respectively. Alignment rates of both mutant and wild-type reads to the reference genome approached 90%. Uniquely mapped reads accounted for 78.0% and 68.0% of the total clean reads in the wild-type and mutant samples, respectively. These results indicate that all experimental procedures—from library construction to sequencing—met the required quality standards.

## 5. Conclusions

In this study, we examined the expression of several PEP-dependent genes (*psbA*, *psbH*, *psaJ*, *trnV*, and *rbcL*) and NEP-dependent genes (*rpoB* and *clpP*). The results indicated a consistent downregulation of PEP-dependent genes in the mutant. By contrast, other NEP-dependent genes were upregulated, aligning with previously documented expression patterns in similar mutants. Both phenotypic assessment and transcript profiling suggest that the loss of PEP function is a primary factor underlying the mutant phenotype. The BSR-Seq approach was employed to map the mutant gene, and the candidate gene was localized to a genomic interval of approximately 900 kb (13.0–13.9) on chromosome 5 of maize. These results are consistent with established models of chloroplast transcriptional regulation in response to mutagenesis and contribute to a deeper understanding of the complex regulatory networks that coordinate chloroplast gene expression.

## Figures and Tables

**Table 1:** Primers used for PCR-based co-segregation analysis.

Forward Primer		Reverse Primer	
57F	ATGTGCATGCCAACCACAGGGT	554R	ATGCGCTCGGCAATGTCCAGTA
533F	TACTGGACATTGCCGAGCGCAT	1015R	AAAAACGGTTCGTCCTGGCCGT
907F	TTCCGTGAGGACGCATTGACCGA	1342R	ATGGGTTGCGATGGTCGTCTTGC
1320F	GCAAGACGACCATCGCAACCCAT	1894R	TTTAATTTCAGCGGCCAGCGCCT
1085F	AACCGTGCGGCTGCATGAAA	1566R	TTGATCGCGGACACAGCCAAGT
1678F	ACAAGCGGCCTTTGTCGTGT	2090R	TGGCGTACCGCGTACATCTTCA
1872F	AGGCGCTGGCCGCTGAAATTAAA	2654R	ACGCTTCGACAGACGGAAAACGG
2349F	AAACCATCCGGCCCGGTACAAA	2897R	TTTCTGCTTTCCGCCATCGGCT
2894F	GAAAGACGACCTGGTAGAAACCT	3491R	GTACGTGCTATCCACAGGAAAGA
2927F	AAACACCACGCACGTTGCCA	3292R	AGCTTGCGCACGGTGAAACA
3205F	CAGAAGCCAGATGGTTGTTCAAG	4148R	GCGGTATTTTCTCCTTACGCATC
3903F	AAACCTCTGACACATGCAGCTCCC	4333R	AGCAACGCGGCCTTTTTACGGT
4160F	TTCCGCTTCCTCGCTCACTGACT	4581R	TGGAGCGAACGACCTACACCGAA
4421F	AAGATACCAGGCGTTTCCCCCT	4868R	AGCGGTGGTTTGTTTGCCGGAT
4421F	AAGATACCAGGCGTTTCCCCCT	5465R	TGCGGAGTGCATCAGGCTCTTT
5237F	CAGGTCGCCGTGGGAAAAGACAA	5674R	ACGGACAGCCGGTATAAAGGGACC
5055F	TCCCAATCAGGCTTGATCCCCAGT	5465R	TGCGGAGTGCATCAGGCTCTTT
5550F	TTGCTCCAGCCATCATGCCGTT	6019R	ACAGAGCGTTGCTGCCTGTGAT
6001F	ACAGGCAGCAACGCTCTGTCAT	6589R	TGGGTTTCTGGCAGCTGGACTT
6574F	AGCTGCCAGAAACCCACGTCAT	6960R	AGACAAGCACGGTCAACTTCCGT
6736F	TTCAGCAGGTGGGTGTAGAGCGT	7635R	TGCCGACAGTGGTCCCAAAGATG
7051F	GGGCGTCGTTCTGGGCTCAT	7969R	CGCTCACTGCCCGCTTTCCA
7407F	TGGGCAATGGAATCCGAGGAGGT	8184R	CGCAAGACCGGCAACAGGATTCA
8037F	CGTATGTTGTGTGGAATTGTGAG	8649R	CAGACATCATCGGTATCAGTGAA
8386F	ATCGCGCGCGGTGTCATCTA	8785R	GCTGCATCTACAGGAGTGGGAGGT
8592F	CCGCTACTGATAGAACCTGCACCG	9150R	ACAGTGTGCCACCTCGTGAAGGA
9134F	ACGAGGTGGCACACTGTTGTCC	9540R	TCAACGTTATGGAGAGCAGCGCA
9352F	GCTCCTGGTTAGCCTCTCTTCCGT	9768R	ACCGTGGACTCAACAACCTTCCT
9555F	GCAACAGACTGAAGCTGTGGGT	10035R	ACCCAGCAACCAGGACCAGAGT
10014F	ACTCTGGTCCTGGTTGCTGGGT	10484R	CGATGCTCACCCTGTTGTTTGGTGT
10474F	GGTGAGCATCGACAAAAGAAACA	11131R	TTTGTCGGGTCATCTTTTCATGC
11120F	TGACCCGACAAACAAGTGCACGG	11815R	TCGACGCAGTCTAACGGACACCA
11815F	ACGGCGTTTAACAGGCTGGCATT	12609R	TAGCATTCGCCATTCAGGCTGCG
12584F	TTGCGCAGCCTGAATGGCGAAT	384R	GTGCAGTTCGGCCCGTTGGT

## Data Availability

Data from this study can be found in the article and Supplementary Materials.

## Supplementary Materials

The following supporting information can be downloaded at: https://www.mdpi.com/article/10.3390/plants15020266/s1, Table S1: The sequence of vector PCAMBIA3300-cry1c.
