# Supplementary material for: Identification of a Chlorophyll-Deficient Mutant in Maize Associated with Exogenous Vector Insertion
Source: Plants (Basel). 2026 Jan 15;15(2):266. doi: 10.3390/plants15020266 (PMC12844738; doi:10.3390/plants15020266)
Supplement: Supplementary file 1 [file plants-15-00266-s001.zip › plants-3943841-supplementary.pdf]

**Supplementary Data**

# Identification of a Chlorophyll Deficient Mutant in Maize Associated with Exogenous Vector Insertion

**WenQi Zhou†*, HaoYue Wang†, ChunXia Liang, HaiJun He, YongSheng Li, XiaoRong Lian, XiaoJuan Wang, XiaoYun Dong, ZengKe Ma, Zhongxiang Liu, YuQian Zhou***

[1] Maize Research Center of Gansu Province, Crop Research Institute, Gansu Academy of Agricultural Sciences, Lanzhou 730070, China

† Equivalent contribution

* Correspondence: zhouwq@gsagr.cn (W.Z.); zhouyuqian@gsagr.cn (Z, Q.)

**Supplementary Table S1** The sequence of vector PCAMBIA3300-cry1c

| Region | Gene/elements | Ori. |
|---|---|---|
| 1041−2041 | pVS1 sta | − |
| 2634−3634 | pVS1 rep | − |
| 4044−4304 | pBR322 bom site | − |
| 4624−4724 | pBR322ori | − |
| 5015−5809 | kanamycin(R) | − |
| 6234−6260 | T−DNA left border(LB) | + |
| 6517−7068 | glufosinate tolerance (bar) / phosphinothricin | − |
| 7081−7850 | cauliflower mosaic virus 35S promoter | − |
| 8109−10408 | Cry1C | − |
| 10409−12447 | Ubiquitin promoter | − |
| 12442−12629 | Lac Z alpha fragment | + |
| 12689−12714 | T−DNA right border(RB) | − |

1 TTAGAATAAC GGATATTTAA AAGGGCGTGA AAAGGTTTAT CCGTTCGTCC ATTTGTATGT

61 GCATGCCAAC CACAGGGTTC CCCTCGGGAT CAAAGTACTT TGATCCAACC CCTCCGCTGC

121 TATAGTGCAG TCGGCTTCTG ACGTTCAGTG CAGCCGTCTT CTGAAAACGA CATGTCGCAC

181 AAGTCCTAAG TTACGCGACA GGCTGCCGCC CTGCCCTTTT CCTGGCGTTT TCTTGTCGCG

241 TGTTTTAGTC GCATAAAGTA GAATACTTGC GACTAGAACC GGAGACATTA CGCCATGAAC

301 AAGAGCGCCG CCGCTGGCCT GCTGGGCTAT GCCCGCGTCA GCACCGACGA CCAGGACTTG

361 ACCAACCAAC GGGCCGAACT GCACGCGGCC GGCTGCACCA AGCTGTTTTC CGAGAAGATC

421 ACCGGCACCA GGCGCGACCG CCCGGAGCTG GCCAGGATGC TTGACCACCT ACGCCCTGGC

481 GACGTTGTGA CAGTGACCAG GCTAGACCGC CTGGCCCGCA GCACCCGCGA CCTACTGGAC

541 ATTGCCGAGC GCATCCAGGA GGCCGGCGCG GGCCTGCGTA GCCTGGCAGA GCCGTGGGCC

601 GACACCACCA CGCCGGCCGG CCGCATGGTG TTGACCGTGT TCGCCGGCAT TGCCGAGTTC

661 GAGCGTTCCC TAATCATCGA CCGCACCCGG AGCGGGCGCG AGGCCGCCAA GGCCCGAGGC

721 GTGAAGTTTG GCCCCCGCCC TACCCTCACC CCGGCACAGA TCGCGCACGC CCGCGAGCTG

781 ATCGACCAGG AAGGCCGCAC CGTGAAAGAG GCGGCTGCAC TGCTTGGCGT GCATCGCTCG

841 ACCCTGTACC GCGCACTTGA GCGCAGCGAG GAAGTGACGC CCACCGAGGC CAGGCGGCGC

901 GGTGCCTTCC GTGAGGACGC ATTGACCGAG GCCGACGCCC TGGCGGCCGC CGAGAATGAA

961 CGCCAAGAGG AACAAGCATG AAACCGCACC AGGACGGCCA GGACGAACCG TTTTTCATTA
1021 CCGAAGAGAT CGAGGCGGAG ATGATCGCGG CCGGGTACGT GTTCGAGCCG CCCGCGCACG
1081 TCTCAACCGT GCGGCTGCAT GAAATCCTGG CCGGTTTGTC TGATGCCAAG CTGGCGGCCT
1141 GGCCGGCCAG CTTGGCCGCT GAAGAAACCG AGCGCCGCCG TCTAAAAAGG TGATGTGTAT
1201 TTGAGTAAAA CAGCTTGCGT CATGCGGTCG CTGCGTATAT GATGCGATGA GTAAATAAAC
1261 AAATACGCAA GGGGAACGCA TGAAGGTTAT CGCTGTACTT AACCAGAAAG GCGGGTCAGG
1321 CAAGACGACC ATCGCAACCC ATCTAGCCCG CGCCCTGCAA CTCGCCGGGG CCGATGTTCT
1381 GTTAGTCGAT TCCGATCCCC AGGGCAGTGC CCGCGATTGG GCGGCCGTGC GGGAAGATCA
1441 ACCGCTAACC GTTGTCGGCA TCGACCGCCC GACGATTGAC CGCGACGTGA AGGCCATCGG
1501 CCGGCGCGAC TTCGTAGTGA TCGACGGAGC GCCCCAGGCG GCGGACTTGG CTGTGTCCGC
1561 GATCAAGGCA GCCGACTTCG TGCTGATTCC GGTGCAGCCA AGCCCTTACG ACATATGGGC
1621 CACCGCCGAC CTGGTGGAGC TGGTTAAGCA GCGCATTGAG GTCACGGATG GAAGGCTACA
1681 AGCGGCCTTT GTCGTGTCGC GGGCGATCAA AGGCACGCGC ATCGGCGGTG AGGTTGCCGA
1741 GGCGCTGGCC GGGTACGAGC TGCCCATTCT TGAGTCCCGT ATCACGCAGC GCGTGAGCTA
1801 CCCAGGCACT GCCGCCGCCG GCACAACCGT TCTTGAATCA GAACCCGAGG GCGACGCTGC
1861 CCGCGAGGTC CAGGCGCTGG CCGCTGAAAT TAAATCAAAA CTCATTTGAG TTAATGAGGT
1921 AAAGAGAAAA TGAGCAAAAG CACAAACACG CTAAGTGCCG GCCGTCCGAG CGCACGCAGC
1981 AGCAAGGCTG CAACGTTGGC CAGCCTGGCA GACACGCCAG CCATGAAGCG GGTCAACTTT
2041 CAGTTGCCGG CGGAGGATCA CACCAAGCTG AAGATGTACG CGGTACGCCA AGGCAAGACC
2101 ATTACCGAGC TGCTATCTGA ATACATCGCG CAGCTACCAG AGTAAATGAG CAAATGAATA
2161 AATGAGTAGA TGAATTTTAG CGGCTAAAGG AGGCGGCATG GAAAATCAAG AACAACCAGG
2221 CACCGACGCC GTGGAATGCC CCATGTGTGG AGGAACGGGC GGTTGGCCAG GCGTAAGCGG
2281 CTGGGTTGTC TGCCGGCCCT GCAATGGCAC TGGAACCCCC AAGCCCGAGG AATCGGCGTG
2341 ACGGTCGCAA ACCATCCGGC CCGGTACAAA TCGGCGCGGC GCTGGGTGAT GACCTGGTGG
2401 AGAAGTTGAA GGCCGCGCAG GCCGCCCAGC GGCAACGCAT CGAGGCAGAA GCACGCCCCG
2461 GTGAATCGTG GCAAGCGGCC GCTGATCGAA TCCGCAAAGA ATCCCGGCAA CCGCCGGCAG
2521 CCGGTGCGCC GTCGATTAGG AAGCCGCCCA AGGGCGACGA GCAACCAGAT TTTTTCGTTC
2581 CGATGCTCTA TGACGTGGGC ACCCGCGATA GTCGCAGCAT CATGGACGTG GCCGTTTTCC
2641 GTCTGTCGAA GCGTGACCGA CGAGCTGGCG AGGTGATCCG CTACGAGCTT CCAGACGGGC
2701 ACGTAGAGGT TTCCGCAGGG CCGGCCGGCA TGGCCAGTGT GTGGGATTAC GACCTGGTAC
2761 TGATGGCGGT TTCCCATCTA ACCGAATCCA TGAACCGATA CCGGGAAGGG AAGGGAGACA
2821 AGCCCGGCCG CGTGTTCCGT CCACACGTTG CGGACGTACT CAAGTTCTGC CGGCGAGCCG
2881 ATGGCGGAAA GCAGAAAGAC GACCTGGTAG AAACCTGCAT TCGGTTAAAC ACCACGCACG
2941 TTGCCATGCA GCGTACGAAG AAGGCCAAGA ACGGCCGCCT GGTGACGGTA TCCGAGGGTG
3001 AAGCCTTGAT TAGCCGCTAC AAGATCGTAA AGAGCGAAAC CGGGCGGCCG GAGTACATCG
3061 AGATCGAGCT AGCTGATTGG ATGTACCGCG AGATCACAGA AGGCAAGAAC CCGGACGTGC
3121 TGACGGTTCA CCCCGATTAC TTTTTGATCG ATCCCGGCAT CGGCCGTTTT CTCTACCGCC
3181 TGGCACGCCG CGCCGCAGGC AAGGCAGAAG CCAGATGGTT GTTCAAGACG ATCTACGAAC
3241 GCAGTGGCAG CGCCGGAGAG TTCAAGAAGT TCTGTTTCAC CGTGCGCAAG CTGATCGGGT
3301 CAAATGACCT GCCGGAGTAC GATTTGAAGG AGGAGGCGGG GCAGGCTGGC CCGATCCTAG
3361 TCATGCGCTA CCGCAACCTG ATCGAGGGCG AAGCATCCGC CGGTTCCTAA TGTACGGAGC
3421 AGATGCTAGG GCAAATTGCC CTAGCAGGGG AAAAAGGTCG AAAAGGTCTC TTTCCTGTGG
3481 ATAGCACGTA CATTGGGAAC CCAAAGCCGT ACATTGGGAA CCGGAACCCG TACATTGGGA
3541 ACCCAAAGCC GTACATTGGG AACCGGTCAC ACATGTAAGT GACTGATATA AAAGAGAAAA

3601 AAGGCGATTT TTCCGCCTAA AACTCTTTAA AACTTATTAA AACTCTTAAA ACCCGCCTGG
3661 CCTGTGCATA ACTGTCTGGC CAGCGCACAG CCGAAGAGCT GCAAAAAGCG CCTACCCTTC
3721 GGTCGCTGCG CTCCCTACGC CCCGCCGCTT CGCGTCGGCC TATCGCGGCC GCTGGCCGCT
3781 CAAAAATGGC TGGCCTACGG CCAGGCAATC TACCAGGGCG CGGACAAGCC GCGCCGTCGC
3841 CACTCGACCG CCGGCGCCCA CATCAAGGCA CCCTGCCTCG CGCGTTTCGG TGATGACGGT
3901 GAAAACCTCT GACACATGCA GCTCCCGGAG ACGGTCACAG CTTGTCTGTA AGCGGATGCC
3961 GGGAGCAGAC AAGCCCGTCA GGGCGCGTCA GCGGGTGTTG GCGGGTGTCG GGGCGCAGCC
4021 ATGACCCAGT CACGTAGCGA TAGCGGAGTG TATACTGGCT TAACTATGCG GCATCAGAGC
4081 AGATTGTACT GAGAGTGCAC CATATGCGGT GTGAAATACC GCACAGATGC GTAAGGAGAA
4141 AATACCGCAT CAGGCGCTCT TCCGCTTCCT CGCTCACTGA CTCGCTGCGC TCGGTCGTTC
4201 GGCTGCGGCG AGCGGTATCA GCTCACTCAA AGGCGGTAAT ACGGTTATCC ACAGAATCAG
4261 GGGATAACGC AGGAAAGAAC ATGTGAGCAA AAGGCCAGCA AAAGGCCAGG AACCGTAAAA
4321 AGGCCGCGTT GCTGGCGTTT TTCCATAGGC TCCGCCCCCC TGACGAGCAT CACAAAAATC
4381 GACGCTCAAG TCAGAGGTGG CGAAACCCGA CAGGACTATA AAGATACCAG GCGTTTCCCC
4441 CTGGAAGCTC CCTCGTGCGC TCTCCTGTTC CGACCCTGCC GCTTACCGGA TACCTGTCCG
4501 CCTTTCTCCC TTCGGGAAGC GTGGCGCTTT CTCATAGCTC ACGCTGTAGG TATCTCAGTT
4561 CGGTGTAGGT CGTTCGCTCC AAGCTGGGCT GTGTGCACGA ACCCCCCGTT CAGCCCGACC
4621 GCTGCGCCTT ATCCGGTAAC TATCGTCTTG AGTCCAACCC GGTAAGACAC GACTTATCGC
4681 CACTGGCAGC AGCCACTGGT AACAGGATTA GCAGAGCGAG GTATGTAGGC GGTGCTACAG
4741 AGTTCTTGAA GTGGTGGCCT AACTACGGCT ACACTAGAAG GACAGTATTT GGTATCTGCG
4801 CTCTGCTGAA GCCAGTTACC TTCGGAAAAA GAGTTGGTAG CTCTTGATCC GGCAAACAAA
4861 CCACCGCTGG TAGCGGTGGT TTTTTTGTTT GCAAGCAGCA GATTACGCGC AGAAAAAAAG
4921 GATCTCAAGA AGATCCTTTG ATCTTTTCTA CGGGGTCTGA CGCTCAGTGG AACGAAAACT
4981 CACGTTAAGG GATTTTGGTC ATGCATTCTA GGTACTAAAA CAATTCATCC AGTAAAATAT
5041 AATATTTTAT TTTCTCCCAA TCAGGCTTGA TCCCCAGTAA GTCAAAAAAT AGCTCGACAT
5101 ACTGTTCTTC CCCGATATCC TCCCTGATCG ACCGGACGCA GAAGGCAATG TCATACCACT
5161 TGTCCGCCCT GCCGCTTCTC CCAAGATCAA TAAAGCCACT TACTTTGCCA TCTTTCACAA
5221 AGATGTTGCT GTCTCCCAGG TCGCCGTGGG AAAAGACAAG TTCCTCTTCG GGCTTTTCCG
5281 TCTTTAAAAA ATCATACAGC TCGCGCGGAT CTTTAAATGG AGTGTCTTCT TCCCAGTTTT
5341 CGCAATCCAC ATCGGCCAGA TCGTTATTCA GTAAGTAATC CAATTCGGCT AAGCGGCTGT
5401 CTAAGCTATT CGTATAGGGA CAATCCGATA TGTCGATGGA GTGAAAGAGC CTGATGCACT
5461 CCGCATACAG CTCGATAATC TTTTCAGGGC TTTGTTCATC TTCATACTCT TCCGAGCAAA
5521 GGACGCCATC GGCCTCACTC ATGAGCAGAT TGCTCCAGCC ATCATGCCGT TCAAAGTGCA
5581 GGACCTTTGG AACAGGCAGC TTTCCTTCCA GCCATAGCAT CATGTCCTTT TCCCGTTCCA
5641 CATCATAGGT GGTCCCTTTA TACCGGCTGT CCGTCATTTT TAAATATAGG TTTTCATTTT
5701 CTCCCACCAG CTTATATACC TTAGCAGGAG ACATTCCTTC CGTATCTTTT ACGCAGCGGT
5761 ATTTTTCGAT CAGTTTTTTC AATTCCGGTG ATATTCTCAT TTTAGCCATT TATTATTTCC
5821 TTCCTCTTTT CTACAGTATT TAAAGATACC CCAAGAAGCT AATTATAACA AGACGAACTC
5881 CAATTCACTG TTCCTTGCAT TCTAAAACCT TAAATACCAG AAAACAGCTT TTTCAAAGTT
5941 GTTTTCAAAG TTGGCGTATA ACATAGTATC GACGGAGCCG ATTTTGAAAC CGCGGTGATC
6001 ACAGGCAGCA ACGCTCTGTC ATCGTTACAA TCAACATGCT ACCCTCCGCG AGATCATCCG
6061 TGTTTCAAAC CCGGCAGCTT AGTTGCCGTT CTTCCGAATA GCATCGGTAA CATGAGCAAA
6121 GTCTGCCGCC TTACAACGGC TCTCCCGCTG ACGCCGTCCC GGACTGATGG GCTGCCTGTA
6181 TCGAGTGGTG ATTTTGTGCC GAGCTGCCGG TCGGGGAGCT GTTGGCTGGC TGGTGGCAGG

6241 ATATATTGTG GTGTAAACAA ATTGACGCTT AGACAACTTA ATAACACATT GCGGACGTTT

6301 TTAATGTACT GAATTAACGC CGAATTAATT CGGGGGATCT GGATTTTAGT ACTGGATTTT

6361 GGTTTTAGGA ATTAGAAATT TTATTGATAG AAGTATTTTA CAAATACAAA TACATACTAA

6421 GGGTTTCTTA TATGCTCAAC ACATGAGCGA AACCCTATAG GAACCCTAAT TCCCTTATCT

6481 GGGAACTACT CACACATTAT TATGGAGAAA CTCGACCCTC AAATCTCGGT GACGGGCAGG

6541 ACCGGACGGG GCGGTACCGG CAGGCTGAAG TCCAGCTGCC AGAAACCCAC GTCATGCCAG

6601 TTCCCGTGCT TGAAGCCGGC CGCCCGCAGC ATGCCGCGGG GGGCATATCC GAGCGCCTCG

6661 TGCATGCGCA CGCTCGGGTC GTTGGGCAGC CCGATGACAG CGACCACGCT CTTGAAGCCC

6721 TGTGCCTCCA GGGACTTCAG CAGGTGGGTG TAGAGCGTGG AGCCCAGTCC CGTCCGCTGG

6781 TGGCGGGGGG AGACGTACAC GGTCGACTCG GCCGTCCAGT CGTAGGCGTT GCGTGCCTTC

6841 CAGGGGCCCG CGTAGGCGAT GCCGGCGACC TCGCCGTCCA CCTCGGCGAC GAGCCAGGGA

6901 TAGCGCTCCC GCAGACGGAC GAGGTCGTCC GTCCACTCCT GCGGTTCCTG CGGCTCGGTA

6961 CGGAAGTTGA CCGTGCTTGT CTCGATGTAG TGGTTGACGA TGGTGCAGAC CGCCGGCATG

7021 TCCGCCTCGG TGGCACGGCG GATGTCGGCC GGGCGTCGTT CTGGGCTCAT GGGTCGAGAG

7081 AGATAGATTT GTAGAGAGAG ACTGGTGATT TCAGCGTGTC CTCTCCAAAT GAAATGAACT

7141 TCCTTATATA GAGGAAGGTC TTGCGAAGGA TAGTGGGATT GTGCGTCATC CCTTACGTCA

7201 GTGGAGATAT CACATCAATC CACTTGCTTT GAAGACGTGG TTGGAACGTC TTCTTTTTCC

7261 ACGATGCTCC TCGTGGGTGG GGGTCCATCT TTGGGACCAC TGTCGGCAGA GGCATCTTGA

7321 ACGATAGCCT TTCCTTTATC GCAATGATGG CATTTGTAGG TGCCACCTTC CTTTTCTACT

7381 GTCCTTTTGA TGAAGTGACA GATAGCTGGG CAATGGAATC CGAGGAGGTT TCCCGATATT

7441 ACCCTTTGTT GAAAAGTCTC AATAGCCCTT TGGTCTTCTG AGACTGTATC TTTGATATTC

7501 TTGGAGTAGA CGAGAGTGTC GTGCTCCACC ATGTTATCAC ATCAATCCAC TTGCTTTGAA

7561 GACGTGGTTG GAACGTCTTC TTTTTCCACG ATGCTCCTCG TGGGTGGGGG TCCATCTTTG

7621 GGACCACTGT CGGCAGAGGC ATCTTGAACG ATAGCCTTTC CTTTATCGCA ATGATGGCAT

7681 TTGTAGGTGC CACCTTCCTT TTCTACTGTC CTTTTGATGA AGTGACAGAT AGCTGGGCAA

7741 TGGAATCCGA GGAGGTTTCC CGATATTACC CTTTGTTGAA AAGTCTCAAT AGCCCTTTGG

7801 TCTTCTGAGA CTGTATCTTT GATATTCTTG GAGTAGACGA GAGTGTCGTG CTCCACCATG

7861 TTGGCAAGCT GCTCTAGCCA ATACGCAAAC CGCCTCTCCC CGCGCGTTGG CCGATTCATT

7921 AATGCAGCTG GCACGACAGG TTTCCCGACT GGAAAGCGGG CAGTGAGCGC AACGCAATTA

7981 ATGTGAGTTA GCTCACTCAT TAGGCACCCC AGGCTTTACA CTTTATGCTT CCGGCTCGTA

8041 TGTTGTGTGG AATTGTGAGC GGATAACAAT TTCACACAGG AAACAGCTAT GACCATGATT

8101 ACGAATTCGA GCTCGAATTT CCCCGATCGT TCAAACATTT GGCAATAAAG TTTCTTAAGA

8161 TTGAATCCTG TTGCCGGTCT TGCGATGATT ATCATATAAT TTCTGTTGAA TTACGTTAAG

8221 CATGTAATAA TTAACATGTA ATGCACAGAT AGGCCTAACG CTTGTCCAAG ATCTATTCAG

8281 GTGCATGACG TTATTTATGA GATGGGTTTT TATGATTAGA GTCCCGCAAT TATACATTTA

8341 ATACGCGATA GAAAACAAAA TATAGCGCGC AAACTAGGAT AAATTATCGC GCGCGGTGTC

8401 ATCTATGTTA CTAGATCGGG AATTCGTTTT TATTTATTTT TTGTCATTAA CGGGTATAAG

8461 CTTTTAATTT CGACTCATCT ATTTTCTGAT ATAAATACGT TGGTCACTAC TTTTGTGCTC

8521 TTTCAAGGTC AGATTCTGCT TCAAATGTTG CATCTGCAAG GATGATCTCG ATCTTGTCGA

8581 TGTAAAGTTC ACCGCTACTG ATAGAACCTG CACCGAAGAG AGGTTGTTCA CTGATACCGA

8641 TGATGTCTGG ATTAGCTCTG AATGAGAAAG GATTACTGAA GTCGGTGTAT CTGAATGTTC

8701 TAGATGTGAG GTTCTCTCCG ATCTCCATAG TTTTCTGAAG AGGCATGTTC ACACTAACTT

8761 GACCTCCCAC TCCTGTAGAT GCAGCTCCTG TAAGAACGAT AACTCGTGCA TCCCTACTAG

8821 AAGCGTAACG AAATCTAAGA CGGTATCTTT GGGTGATTGG TGAGTTGATG TTCACTTGAA

8881 GAGAAACGAA GTCACCAAAG GTGTTTCTTC GAAGGATATC ACCTCCTGTG AATCCTGGTC
8941 CTGTAATCAC AGAGGTTCCT CCCCAAACTC TGAATCCTTT CACAAGAGGG ATCTGGTTGA
9001 TCCTCTCTGG ATCAATTGTG TTGGTAAGAG TTGCACTACG ATGAGTCCAA GAGAACACAA
9061 CACCAGTTGT AAGGAAAGGT GTTCCAGATC TTTGAACGAA GGTTGCATGA CAAAGACGAT
9121 GACTGTATCC TTCACGAGGT GGCACACTGT TGTCCTCAGG TGGAAGTTCA GTAAGAGAAT
9181 CAACAGTACC TCTTCCACGA TAGGTGAAGC TGTTTGTAGG TGTAGAGAAC TCAACTCCTT
9241 CAACACCACG AAGGTTGAAT GGTGGAGCTG GCCAAGGTTG CTGAAGAAGT CGAAGAGTAG
9301 GATTTGAAAG AGTCCTGAAC ACAGGTCCGT TGAAAGTGAA TGATCTTGGA GGCTCCTGGT
9361 TAGCCTCTCT TCCGTAGATA GGAGATGTGA TGTTACCACC TCCGATGAGG CTAGAGATCA
9421 GTCGATGTCC TCCCCAGTAG AAGTTACGTC CAACACTAAA CCAATCGGTA AAGATTGTAA
9481 GGTTGTTCAA GATGTCGAAG AGGTGAGGAT TTCTGATTGC GCTGCTCTCC ATAACGTTGA
9541 AGGTAGGAAG CTGAGCAACA GACTGAAGCT GTGGGTTGAA GTTGATGAGT GGGTCAGTGT
9601 AAACTTCCCT TGTAAGTTGA CCAACTGGCT GAATTGGATA TCTCCTATTG TCATAGTTTG
9661 GAAAGAAAGC AGCGATATCA AGAACAGTCA ATGTAAGGTC TCTCCTAAGT CGGTTGTATG
9721 TGATCCAATC TTGATAGGTA GACTTAGGAA GGTTGTTGAG TCCACGGTTG TAAGTGTTAG
9781 CACAGTGATC AGCGTACTCA TCGATGTGCC TGATGAGTCT GTTGTAGTTC TCGTTCACGT
9841 TGATGGTTGT CAATCCCCAT CTTTCTCCGA AGATCACAGA ATCTCTAAGG ATAGCAAGAT
9901 GGAGATTAGC AGCTTGAGCG TAAACAGAGA GAAGTGGAAC TTCAAATCCA GAGATCCTAA
9961 AGCTAGGAAT GTCCCTTTCA AGAAGTCCAT CAAGGATACG GAACCTATCG ATCACTCTGG
10021 TCCTGGTTGC TGGGTTGTTA GGATCTTCTT CCCATTCCTT GAATGCCTCC ACGTAGATGT
10081 TGAAGTTGTT TCCAAGTCCT TCAAGGTTAG CAATAGCAGC ATTCCTAGCG AACTCAGCGA
10141 TTCTTTCGTT GATGAGCTGT TCGATCTGAA CGAGAAATGC ATCCCATTGA GAAGGTCCAA
10201 CGATTCCCCA AACGAAGTCG ATAAGTCCAA CAAGGAATCC TCCTCCTGGC ACAAAGTTAG
10261 AAACCAAGAA CTGAACAAGT GAGAGAGAGA TGTCAATTGA TGAGTTACCA GTTGAGATCC
10321 TTTCTCCATC CAAAAGAACT TCTTCAGGAT TAGAAAGACA ATTGTAAGGG ATACACTGGT
10381 TCTGATTGTT CTCCTCCATG GTGGATCCTG ATAGTCTTGC CAGTCAGGGT TTTCACAAAG
10441 ATCTGCATCT GCAGAAGTAA CACCAAACAA CAGGGTGAGC ATCGACAAAA GAAACAGTAC
10501 CAAGCAAATA AATAGCGTAT GAAGGCAGGG CTAAAAAAAT CCACATATAG CTGCTGCATA
10561 TGCCATCATC CAAGTATATC AAGATCAAAA TAATTATAAA ACATACTTGT TTATTATAAT
10621 AGATAGGTAC TCAAGGTTAG AGCATATGAA TAGATGCTGC ATATGCCATC ATGTATATGC
10681 ATCAGTAAAA CCCACATCAA CATGTATACC TATCCTAGAT CGATATTTCC ATCCATCTTA
10741 AACTCGTAAC TATGAAGATG TATGACACAC ACATACAGTT CCAAAATTAA TAAATACACC
10801 AGGTAGTTTG AAACAGTATT CTACTCCGAT CTAGAACGAA TGAACGACCG CCCAACCACA
10861 CCACATCATC ACAACCAAGC GAACAAAAGC ATCTCTGTAT ATGCATCAGT AAAACCCGCA
10921 TCAACATGTA TACCTATCCT AGATCGATAT TTCCATCCAT CATCTTCAAT TCGTAACTAT
10981 GAATATGTAT GGCACACACA TACAGATCCA AAATTAATAA ATCCACCAGG TAGTTTGAAA
11041 CAGAATTCTA CTCCGATCTA GAACGACCGC CCAACCAGAC CACATCATCA CAACCAAGAC
11101 AAAAAAAAGC ATGAAAAGAT GACCCGACAA ACAAGTGCAC GGCATATATT GAAATAAAGG
11161 AAAAGGGCAA ACCAAACCCT ATGCAACGAA ACAAAAAAAA TCATGAAATC GATCCCGTCT
11221 GCGGAACGGC TAGAGCCATC CCAGGATTCC CCAAAGAGAA ACACTGGCAA GTTAGCAATC
11281 AGAACGTGTC TGACGTACAG GTCGCATCCG TGTACGAACG CTAGCAGCAC GGATCTAACA
11341 CAAACACGGA TCTAACACAA ACATGAACAG AAGTAGAACT ACCGGGCCCT AACCATGGAC
11401 CGGAACGCCG ATCTAGAGAA GGTAGAGAGG GGGGGGGAGG ACGAGCGGCG TACCTTGAAG
11461 CGGAGGTGCC GACGGGTGGA TTTGGGGGAG ATCTGGTTGT GTGTGTGTGC GCTCCGAACA

11521 ACACGAGGTT GGGGAAAGAG GGTGTGGAGG GGGTGTCTAT TTATTACGGC GGGCGAGGAA
11581 GGGAAAGCGA AGGAGCGGTG GGAAAGGAAT CCCCCGTAGC TGCCGGTGCC GTGAGAGGAG
11641 GAGGAGGCCG CCTGCCGTGC CGGCTCACGT CTGCCGCTCC GCCACGCAAT TTCTGGATGC
11701 CGACAGCGGA CGAAGTCCGG TACCGAGGCA GCGACAGAGA TGCCGTGCCG TCTGCTTCGC
11761 TTGGCCCGAC GCGACGCTGC TGGTTCGCTG GTTGGTGTCC GTTAGACTGC GTCGACGGCG
11821 TTTAACAGGC TGGCATTATC TACTCGAAAC AAGAAAAATG TTTCCTTAGT TTTTTTAATT
11881 TCTTAAAGGG TATTTGTTTA ATTTTTAGTC ACTTTATTTT ATTCTATTTT ATATCTAAAT
11941 TATTAAATAA AAAAACTAAA ATAGAGTTTT AGTTTTCTTA ATTTAGAGGC TAAAATAGAA
12001 TAAAATAGAT GTACTAAAAA AATTAGTCTA TAAAAACCAT TAACCCTAAA CCCTAAATGG
12061 ATGTACTAAT AAAATGGATG AAGTATTATA TAGGTGAAGC TATTTGCAAA AAAAAAAAGG
12121 AGAACACATG CACACTAAAA AGATAAAACT GTAGAGTCCT GTTGTCAAAA TACCAATTGT
12181 CCTTTAGACC ATGTCTAACT GTTCATTTAT ATGATTCTCT AAAACACTGA TATTATTGTA
12241 GTACTATAGA TTATATTATT CGTAGAGTAA AGTTTAAATA TATGTATAAA GATAGATAAA
12301 CTGCACTTCA AACAAGTGTG ACAAAAAAAA TATGTGGTAA TTTTTTATAA CTTAGACATG
12361 CAATGCTCAT TATCTCTAGA GAGGGGCACG ACCGGGTCAC GCTGCACTGC AGTCTACCTT
12421 GCCATGCCGC CGGATTCAGC TAAGCTTGGC ACTGGCCGTC GTTTTACAAC GTCGTGACTG
12481 GGAAAACCCT GGCGTTACCC AACTTAATCG CCTTGCAGCA CATCCCCCTT TCGCCAGCTG
12541 GCGTAATAGC GAAGAGGCCC GCACCGATCG CCCTTCCCAA CAGTTGCGCA GCCTGAATGG
12601 CGAATGCTAG AGCAGCTTGA GCTTGGATCA GATTGTCGTT TCCCGCCTTC AGTTTAAACT
12661 ATCAGTGTTT GACAGGATAT ATTGGCGGGT AAACCTAAGA GAAAAGAGCG TTTA
